# Analysis of Indications for Voiding Cystography in Children

**DOI:** 10.3390/jcm10245809

**Published:** 2021-12-11

**Authors:** Natalia Kopiczko, Aleksandra Dzik-Sawczuk, Karolina Szwarc, Anna Czyż, Anna Wasilewska

**Affiliations:** Department of Pediatrics and Nephrology, Medical University of Bialystok, ul. Waszyngtona 17, 15-274 Bialystok, Poland; aleksandradzik93@gmail.com (A.D.-S.); karolina.szwarc@interia.pl (K.S.); aczzz@wp.pl (A.C.); annwasil@interia.pl (A.W.)

**Keywords:** voiding cystography, infection, urinary tract

## Abstract

In this study, we report the experience of our center with the prognosis of vesicoureteral reflux, depending on the indications for voiding cystography, during a 12-year period. Retrospective analysis included 4302 children who were analyzed according to the indication for voiding cystography: (1) a febrile urinary tract infection, (2) urinary tract malformations on ultrasonography and (3) lower urinary tract dysfunction. Vesicoureteral reflux was found in 917 patients (21.32%; 24.1% of girls and 17.9% of boys). In group (1), reflux was found in 437/1849 cases (23.63%), group (2) in 324/1388 cases (23.34%) and group (3) in 156/1065 cases (14.65%). A significantly lower prevalence of reflux and its lower degree was found in children from group (3) when compared to other groups (*p* < 0.01). VURs were confirmed in over 20% of children with urinary tract malformations on ultrasonography or after a febrile urinary tract infection, suggesting the need for voiding cystography in these children. Indications for this examination in children with lower urinary tract dysfunction should be limited.

## 1. Introduction

Vesicoureteral reflux (VUR) is defined as a pathological retrograde flow of urine from the bladder to the upper parts of the urinary tract. The prevalence of VUR is still difficult to estimate. In an epidemiological study by Sargent, VUR was found in 0.4% to 1.8% of the general population of children and in over 30% of children after a febrile urinary tract infection (UTI) [1]. Similarly, other authors suggested VUR is a rather uncommon disorder that only affects a low percentage of all children [2]. However, the latest review article on VUR by Tullus, suggested the possibility of a much higher prevalence of VUR in the population (25%) [3]. This conclusion was based on large cohort studies, which reported vesicoureteral reflux in 65% of infants in the first 6 months of life [4]. Similarly, a Finnish group retrospectively diagnosed a large cohort of children who had undergone voiding cystography (VCUG) and confirmed reflux in 28% to 36% of cases, depending on the indication for this examination [5].

Although the percentage of children affected with VUR seems to be high, in recent years, there is a trend towards reducing the number of investigations for this abnormality.

The guidelines from the UK National Institute for Health and Care Excellence and the American Academy of Pediatrics no longer recommend radiological investigations to detect vesicoureteral reflux in most children with an uncomplicated febrile UTI [6,7].

The indications for voiding cystography have been changed over time and usually include: a recent febrile urinary tract infection, especially in neonates and young children; abnormalities in renal and bladder ultrasonogram (RBUS) [6]; lower urinary tract dysfunction (LUTD), if they do not respond to standard therapy [8]. In the recent literature, the follow-up strategy for children after first-time acute pyelonephritis has been debated; however, no consensus has been achieved. It was shown that non-*E. coli* infections and elevated plasma creatinine are predictors of kidney scarring. Additionally, the authors showed that delayed clinical response time (>48 h), non-*E. coli* infection and an abnormal kidney US are significant predictors of an uneven differential kidney function [9]. That is why future work must focus on identifying further risk factors, new imaging modalities or specific urine or blood biomarkers, in order to minimize the number of investigations and identify those at risk for recurrent UTIs and loss of kidney function.

Diagnosis of a UTI in a febrile child requires both a positive urinalysis and urine culture. The method of obtaining a urine specimen is critical for accurate diagnosis. A urine specimen should be obtained either through sterile transurethral catheterization or suprapubic aspiration if a clean-catch specimen is not feasible. The diagnosis of UTI cannot be reliably established by a bagged specimen. [10]

In this study, we report the experience of our center with the prognosis of vesicoureteral reflux, depending on the indications for voiding cystography, during a 12-year period.

## 2. Materials and Methods

We performed a retrospective chart review of all children with VUR who were followed up at the Pediatric Nephrology Department, Medical University of Bialystok, Poland, between January 2003 and December 2014, as the recommendations for voiding cystography were quite wide during this period so we had a lot of patients for analysis. All children with VUR diagnosed with the use of VCUG were included in the study. They were graded using a 5-grade scale proposed by the International Reflux Study in Children [11].

During that time period, 4523 children had undergone voiding cystography (VCUG) in our center. Retrospective analysis included 4302 patients, whose medical charts allowed the determination of their indication for VCUG.

The inclusion criteria were patients aged 0–18 years who had undergone VCUG and whose medical charts were complete. Patients with a neurogenic bladder, myelomeningocele or posterior ureteral valves were excluded from the study. Reflux was also defined as passive (contrast solution is seen after filling the bladder) and active (during urination). The standard term ‘reflux unit’ was used to refer to reflux found on one side of the body. All patients had undergone ultrasonography of their urinary tract. The study group was divided into three subgroups based on their major indication for VCUG: (1) a febrile urinary tract infection (UTI), (2) urinary tract malformations on USG (UTM) and (3) lower urinary tract dysfunction (LUTD).

The definition of a febrile UTI was defined as: a UTI with a fever (above 38.5 °C) and increased inflammatory markers (leukocytosis, increased CRP and increased procalcitonin levels).

As recommendations changed during the analyzed period, the indications for VCUG were additionally assessed in two time intervals: (1) years 2003–2008 and (2) years 2009–2014.

The study had approval from the Local Bioethics Committee at the Medical University of Bialystok. Statistical analysis was performed using Statistica 10.0 software. The sample size was appropriate. The comparison between the groups was carried out with the Chi-square test for categorical variables. Statistical significance was set for *p* < 0.05.

## 3. Results

The median (IQR) age of patients included in the analysis was 4 years (0.8–9.0); 5 years (1.5–9.0) for girls and 3 years (0.5–8.0) for boys. Of the 4302 children who took part in our study, 2360 were females (55%) and 1942 were males (45%). Of the study population, 917 patients (21.3%) were positive for VUR (1266 reflux units). Girls were affected significantly more often than boys (569 vs. 348; *p* < 0.01). The discrepancy between the number of patients diagnosed with VUR and the number of reflux units is because 349 children (38.0%) had bilateral reflux. There were 533 (42.1%) active reflux cases and 733 (57.9%) passive reflux cases. The reflux units were found significantly more often on the left side (61.9%), when compared to the right side (*p* < 0.01). In children with a UTM, reflux was found on the affected side or on both sides—the affected side and additionally on the contralateral side. Out of all grades, the second one was most commonly found and made up 37.6% of the diagnosed reflux units (Table 1).

The most frequent urinary tract malformation found on USG was hydronephrosis, with or without caliectasis, of a mild, moderate and severe degree, which was assessed according to the Society for Fetal Urology and anteroposterior diameter of the renal pelvis grading systems: grade 1 (urine in pelvis barely splits sinus), grade 2 (urine fills intrarenal pelvis ± major calyces dilated) and grade 3 (virtually all calyces are visualized), and grade 4 (similar to grade 3 plus parenchymal thinning) [12]. As we mentioned before, children with a neurogenic bladder, myelomeningocele or posterior ureteral valves were excluded from the study. In the group with a UTM and vesicoureteral reflux, hydronephrosis of grade 1 or 2 was found in 285/324 cases (88%), grade 3 in 33/324 cases (10%) and grade 4 in 6/324 cases (2%). Children who were diagnosed with reflux of grade 4 or 5, had grade 1 or 2 hydronephrosis in 66/114 cases (58%), grade 3 in 29/114 cases (25%) and grade 4 in 19/114 cases (17%). In the group with a UTM who were negative for reflux, most of children had hydronephrosis of grade 1 or 2, but still in about 9% of patients, it was grade 3 or 4.

Most of the children diagnosed with reflux from this group had grade 1 or grade 2 hydronephrosis, in 285/324 cases (88%). The rest of the children with reflux were classified as having grade 3 or grade 4 hydronephrosis.

The rate of VUR diagnosis was the highest in group (1), in children after a UTI (43.0%), lower with a UTM and the lowest with LUTD. The difference was statistically significant in group (3), when compared both to group (1) (*p* < 0.01) and group (2) (*p* < 0.01). No significant difference was observed between the incidence of positive VUR in group (1) and group (2) (*p* > 0.05).

A significantly higher incidence of moderate and severe reflux was found in group (1) and group (2), when compared to group (3) (*p* < 0.01 and *p* < 0.01, respectively). Children with LUTD had the highest incidence of reflux grade 1 and 2. The results are presented in Table 2.

Further analysis included data in two time intervals. A significantly higher number of VCG were performed in the years 2003–2008, when compared to the years 2009–2014 (*p* < 0.01). Interestingly, the percentage of children diagnosed with VUR in the years 2009–2014 was higher (24.2%) than in 2003–2008 (19.2%) (*p* < 0.01). The rate of positive VUR was significantly higher in the years 2009–2014, in children from group (1) and group (3), than in the years 2003–2008 (*p* < 0.01 and *p* < 0.05, respectively). In the years 2003–2008, a UTM found on USG was the indication for VCUG significantly more often than the other two reasons (*p* < 0.01), while in 2009–2014, the most frequent sign was a febrile UTI (Table 3). When the patients had both indications (they had a UTI and additionally presented with a urinary tract malformation), the incidence of a positive examination for reflux was 31.55% and 44.92%, in both time intervals, respectively.

## 4. Discussion

In this study, we evaluated the rate of detection of reflux in the population of children diagnosed in the Pediatric Nephrology Department, Medical University of Bialystok. In the entire study group, the prevalence of reflux was 21.3% and was almost as high as suggested by Tullus [3]. The prevalence of VUR in American children after a febrile UTI was almost twice as high as in our patients; however, this research only included children with a UTI and an age of less than 60 months [13]. Similar results were found in Swedish children with a UTI [14].

The incidence of VUR in children with a UTM on USG in our study was 23.3%, and this shared similarities with research by Brophy et al. [15]. In children with a febrile UTI or a UTM on USG, the incidence of moderate and severe reflux was significantly higher than in the group with LUTD. This may be explained because patients with higher grades of reflux are usually diagnosed earlier, because of symptomatic presentation (a UTI) or abnormal ultrasonography results. The frequency of VUR in children with LUTD in this study was only 14.6% and was similar to Iranian children [16] and much lower than in Japanese patients with enuresis [17]. Although Sillen et al. have indicated that patients who have both VUR and LUTD may have a worse final outcome after treatment, including an elevated risk of kidney damage, there is no indication for performing VCUG in all children with LUTD [18]. The results of the above study indicate that the coexistence of both conditions should be explored in any patient who has VUR, until the patient presents with symptoms suggestive of LUTD (urgency, wetting, constipation or holding maneuvers) and an extensive history, and has an examination, including voiding charts, uroflowmetry and residual urine determination. On the other hand, in LUTD, VUR is often low-grade and ultrasonography findings are often normal. It is recommended to ask all patients with LUTD if they have a history of febrile UTIs, in which case there is a greater possibility of finding VUR. Urodynamic study combined with VCUG is recommended in any child who fails standard therapy for LUTD.

On further analysis, we analyzed the results in two periods, as the recommendations for VCUG in these two periods changed. Previously, it was performed in most children after their first UTI. Similarly, increased pelvis size of the kidney was the indication for this diagnostic examination. Nowadays, we use the following approach, which avoids performing VCUG unnecessarily in most infants with fetal hydronephrosis. Voiding cystography is recommended if there is a bilateral antenatal hydronephrosis in a male or if the postnatal ultrasound shows persistent moderate or severe hydronephrosis (renal pelvic diameter >10 mm) and/or ureteral dilatation. Additionally, this examination should be considered if there is a family history of VUR. [19]. These recommendations are in line with the observation provided in this study. We found that reflux of a high grade (4 and 5) was found in only 114/1388 (8%) of all examined children with a UTM. In UTM group, high grade hydronephrosis was found in 12% of children who were positive for reflux, but also in 9% children who were negative for reflux.

Recently, routine imaging of infants with VCUG after the first UTI is no longer suggested, unless an ultrasonogram suggests selected renal abnormalities or obstruction, or a high-grade VUR. VCUG is usually indicated for children <2 years of age with a second well-documented UTI [7,20]. Starting from the year 2009, the number of VCUG decreased over time, from 2457 in the years 2003–2008 to 1845 in the years 2009–2014. What is even more important is that the accuracy of reflux diagnosis increased notably. This proves more accurate patient selection for this imaging study. Additional analysis showed that girls were diagnosed with VUR more often than boys. Low-grade reflux is often diagnosed during urological diagnostics. Significantly, a higher incidence of moderate and severe reflux was found in the group after a UTI and with a UTM, when compared to the group with LUTD. Analyzing two time intervals, the rate of positive VUR was less than 20% in the years 2003–2008 and increased to almost 25% in 2009–2014, which resulted from more accurate analysis of indications for VCUG.

The strength of this study is the long study period and large number of patients included in the study, analyzed in selected groups, reaching a power-calculated number of participants. Although our population reached a large number, our study still has its limitations. Primarily, it is a retrospective study and we assessed patients according to their major indication for VCUG, while in many children, the coexistence of two, or even three indications might be observed. The classification of a UTI was not always specified as atypical, recurrent or neither. This may influence evaluation of the classification of a UTI as a predictor for abnormal VCUG. A similar situation may apply to children with LUTD, as this diagnosis is sometimes difficult, especially in a retrospective study.

Starting from 2015, according to recommendations, we changed the indications for VCUG, and it is now only proposed for children with a recurrence of pyelonephritis or with significant abnormalities in an ultrasonogram. In the future, we will analyze the results of VCUG according the latest indications. It is in agreement with the results of RIVUR and CUTIE data, published in 2021, where the top-down approach (DMSA—as the first diagnostic study) was associated with a slightly higher recurrent UTI; however, compared to a bottom-up approach (VCUG as the first study), it significantly reduced the need of VCUG [21].

## 5. Conclusions

In our study, the incidence of positive reflux in the years 2003–2008 was the highest in children with abnormal USG, was lower with a febrile UTI and was the lowest with LUTD. However, after the recommendations changed in the years 2009–2014 the incidence of positive refluxes was the highest in UTI group. In conclusion, we would like to point out that the recommendations for voiding cystography in children is still a controversial area, but advances have been made towards less aggressive management than that applied traditionally. VCUG should be strongly considered in children after first-time acute pyelonephritis caused by non-*E. coli* infections, with elevated plasma creatinine, male with a bilateral antenatal hydronephrosis or if the postnatal ultrasound shows persistent moderate or severe hydronephrosis (renal pelvic diameter >10 mm), and/or ureteral dilatation, especially when there is a family history of VUR. Urodynamic study combined with VCUG is also recommended in any child who fails standard therapy for LUTD. The initial imaging approach for children with urinary tract infection, urinary tract malformations and disturbances is controversial, and further analysis is required to propose a strong recommendation in this field.

## Figures and Tables

**Table 1:** Characteristics of examined children.

	*N*	Median Age (Q1–Q3)	Positive Reflux		Reflux Units	Positive Reflux (Unit)		Bilateral Reflux	Unilateral Reflux	
			*N*	%		Active	Passive		Right	Left
Total	4302	4 (0.8–9.0)	917	21.3	1266	533	733	349	210	358
Female	2360	5 (1.5–9.0)	569	24.1	790	318	472	221	122	226
Male	1942	3 (0.5–8.0)	348	17.9	476	215	261	128	88	132

**Table 2:** Grading of positive reflux, depending on indication for VCUG.

Grade (Total)	Urinary Tract Infection	Urinary Tract Malformations on USG	Lower Urinary Tract Dysfunction
1	134 (22%)	99 (23%)	76 (37.5%)
2	239 (39%)	146 (33%)	92 (44%)
3	132 (21%)	81 (18%)	34 (16%)
4	77 (12%)	60 (14%)	5 (2%)
5	36 (6%)	54 (12%)	1 (0.5%)

**Table 3:** Indications for VCG and positive reflux diagnosis in two time intervals (2003–2008 vs. 2009–2014).

	Years 2003–2008 (*n* = 2457)	Years 2009–2014 (*n* = 1845)	*p* Value
Urinary tract Infection	189/974 (19.4%)	248/874 (28.4%)	0.01
Urinary tract malformations on USG	197/814 (24.2%)	127/574 (22.1%)	0.37
Lower urinary tract dysfunction	85/669 (12.7%)	71/396 (17.9%)	0.02
